# Hymenal Tags in Girls: Not to Be Mistaken for Sexual Abuse

**DOI:** 10.7759/cureus.17931

**Published:** 2021-09-13

**Authors:** Alexander K Leung, Joseph M Lam

**Affiliations:** 1 Pediatrics, University of Calgary, Calgary, CAN; 2 Department of Dermatology and Skin Sciences, The University of British Columbia, Vancouver, CAN

**Keywords:** sexual abuse, protruding vaginal mass, hymenal tag, hymenal polyp, hymenal anomalies

## Abstract

Although hymenal tags are not uncommon in newborn girls, there is a paucity of literature on this condition. Few photo images of hymenal tags have been published. We report the case of a four-month-old girl with a large hymenal tag noted at birth. Approximately 30% of hymenal tags may persist at three years of age. As the number of prepubertal girls who require evaluation for sexual abuse increases, physicians should familiarize themselves with the appearance of a hymenal tag to differentiate it from signs of sexual abuse, especially in older girls whose hymenal tags have not resolved.

## Introduction

Hymenal tags, also known as hymenal polyps, are common findings in infant girls. Despite its prevalence, there is a paucity of literature on hymenal tags. A PubMed search of the English literature conducted in April 2021 using the key terms “hymenal tag” OR “hymenal polyp” revealed only seven publications [1-7]. The majority of hymenal tags are small, and hymenal tags of considerable size are unusual. Although hymenal tags tend to resolve over time, approximately 30% may persist at three years of age [4]. The persistence of hymenal tags in an older girl may lead to the suspicion of sexual abuse. Herein, we report the case of a four-month-old girl with a large hymenal tag. The purpose of this communication is to alert physicians that the persistence of a hymenal tag in an older girl should not be mistaken for a torn hymen, which is a sign of sexual abuse.

## Case presentation

A four-month-old Chinese infant girl presented with a protruding vaginal mass which was first noted at birth. The infant was born to a gravida 2 para 1 27-year-old mother at 39 weeks of gestation, following an uncomplicated pregnancy and normal vaginal delivery. The mother was not on any medications during the pregnancy. The Apgar scores were 6 and 9 at one and five minutes, respectively. Her birth weight was 3.02 kg (between 25th and 50th centile), length was 51.1 cm (between 50th and 75th centile), and head circumference was 35.4 cm (75th centile). The labor was smooth and there was no history of birth trauma or vaginal bleeding. The infant was exclusively breasted and thriving. The mass was asymptomatic and there was no history of bleeding from the mass or of sexual abuse. The infant came from a loving and supportive family. Her mother was the sole care provider.

**Figure 1:**
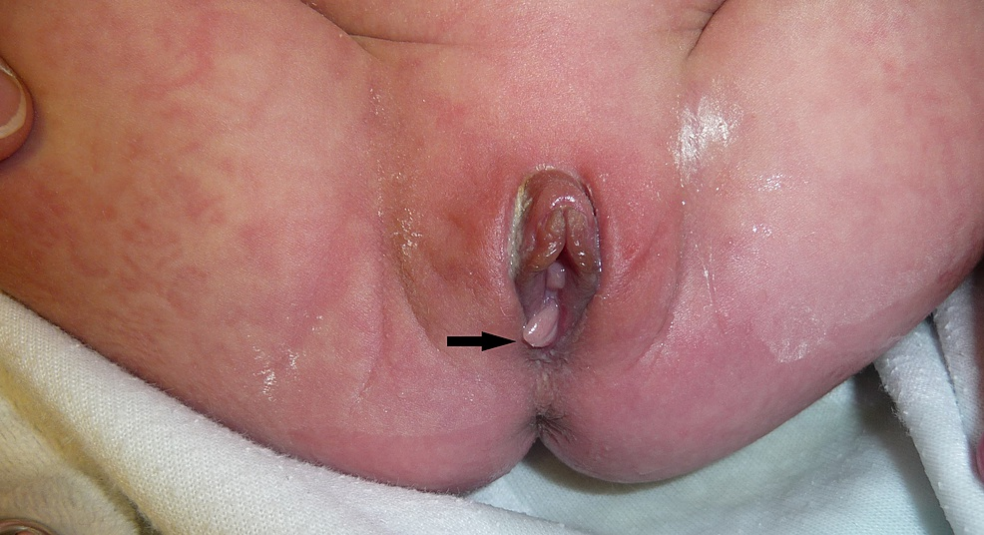
A four-month-old infant noted to have a smooth, pink mass extending from the hymen.

On examination, the infant looked alert and was not in distress. Her weight was 6.8 kg, length was 64 cm, and head circumference was 41.7 cm. A smooth, pink mass was seen extending from the hymenal ring (Figure 1). The mass measured 18 mm long and 5 mm wide. The rest of the physical examination was normal.

Based on the appearance and location of the mass extending from the hymenal ring and its onset during the neonatal period, a diagnosis of a hymenal tag was made. Parents were reassured of the benign nature of this condition and that no treatment was required.

## Discussion

Hymenal tags are not uncommon in newborn girls. However, there have been very few reports and photo images published on this condition. Hymenal tags are often overlooked unless specifically sought for. The incidence of hymenal tags is estimated to range from 6% to 13% of newborn girls [1,7]. In one study, of the 974 female neonates admitted to the newborn department of the Beilinson Medical Center in Israel, 56 (5.75%) neonates were found to have hymenal tags during routine physical examinations within the first 24 hours of life [7]. In another study, 468 female neonates born at the University of Texas Medical Branch in the United States had their genitalia examined during the neonatal period before discharge [1]. Sixty-nine hymenal tags were observed in 59 (13%) neonates, with 10 neonates having two hymenal tags [1]. A prospective study examined the genitalia of 211 girls between one month and seven years of age (mean age of 21 months) who presented for child care or non-gynecologic complaints with no history of sexual abuse and found hymenal tags in six (2.8%) of the girls [2]. The study population consisted of 36% blacks, 33.6% white non-Hispanics, 29.9% Hispanics, and 0.5% Asians.

Clinically, a hymenal tag presents as an elongated projection of hymenal tissue extending from the hymenal rim, intravaginal ridge, or external vestibular hymenal ridge [1,3,6]. The tag is pink in color and has a smooth surface [7]. The condition is usually isolated and solitary. Occasionally, there may be two or more hymenal tags [7].

The majority of hymenal tags are small. Large hymenal tags are unusual. The tags are most commonly found in the superior (ventral) and inferior (dorsal) positions of the hymen and, less commonly, in the lateral position [1,2]. Hymenal tags may represent a remnant of a vaginal septum present earlier in fetal development [8]. New hymenal tags may develop postnatally from cleavage of a congenital hymenal septum or as a result of the extension of an intravaginal ridge or external vestibular hymenal ridge [4,8]. The condition is usually asymptomatic. Occasional complications include bleeding and infection [6,8].

Hymenal tags tend to regress with time [4]. Some authors suggest that the regression is due to aging and the waning effects of estrogen which is relatively high during the neonatal period [4,6,7]. Approximately 70% of hymenal tags have resolved by three years of age [4]. In the study by Berenson, 15 (68%) of the 22 hymenal tags that were present at birth were not observed at three years of age [4]. Parents should be reassured of the benign nature of the condition and that no treatment is necessary. As 30% of hymenal tags may persist at three years of age and the number of prepubertal girls who require evaluation for sexual abuse increases, physicians should be knowledgeable about the appearance of a hymenal tag to differentiate it from a torn hymen which may result from sexual abuse, especially in an older girl whose hymenal tag is of considerable size and has not resolved. Rarely, the hymenal tag may persist into adulthood. Borko et al. reported the case of a 21-year-old female with a persistent hymenal tag who had no prior history of sexual intercourse [5].

## Conclusions

Routine physical examination of children should include a careful examination of the external genitalia. Physicians should be familiar with hymenal tags to diagnose them accurately so that they will not be mistaken for sexual abuse, especially in an older girl with the persistence of a hymenal tag of considerable size.
